# Feasibility of a novel dose fractionation strategy in TMI/TMLI

**DOI:** 10.1186/s13014-018-1201-0

**Published:** 2018-12-17

**Authors:** Zhirong Bao, Hongli Zhao, Dajiang Wang, Jian Gong, Yahua Zhong, Yu Xiong, Di Deng, Conghua Xie, An Liu, Xiaoyong Wang, Hui Liu

**Affiliations:** 1 Department of Radiation and Medical Oncology, Zhongnan Hospital of Wuhan University, Wuhan, Hubei China; 2 Hubei Radiotherapy Quality Control Center, Wuhan University, Wuhan, Hubei China; 3 Divisions of Radiation Oncology, City of Hope National Medical Center, Duarte, CA USA

**Keywords:** TMI/TMLI, Helical Tomotherapy, Radiotherapy, Bone marrow transplantation

## Abstract

**Background:**

To report our experience in planning and delivering total marrow irradiation (TMI) and total marrow and lymphatic irradiation (TMLI) in patients with hematologic malignancies.

**Methods:**

Twenty-seven patients undergoing bone marrow transplantation were treated with TMI/TMLI using Helical Tomotherapy (HT). All skeletal bones exclusion of the mandible comprised the treatment target volume and, for TMLI, lymph node chains, liver, spleen and/or brain were also included according to the clinical indication. Planned dose of 8Gy in 2 fractions was delivered over 1 day for TMI while 10Gy in 2 fractions BID was used for TMLI. Organs at risk (OAR) contoured included the brain, brainstem, lens, eyes, optic nerves, parotids, oral cavity, lungs, heart, liver, kidneys, stomach, small bowel, bladder and rectum. In particular, a simple method to avoid hot or cold doses in the overlapping region was implemented and the plan sum was adopted to evaluate dose inhomogeneity. Furthermore, setup errors from 54 treatments were summarized to gauge the effectiveness of immobilization.

**Results:**

During the TMI/TMLI treatment, no acute adverse effects occurred during the radiation treatment. Two patients suffered nausea or vomiting right after radiation course. For the 9 patients treated with TMI, the median dose reduction of major organs varied 30–65% of the prescribed dose, substantially lower than the traditional total body irradiation (TBI). Meanwhile, average biological equivalent doses to OARs with 8Gy/2F TMI approach were not different from the conventional 12Gy/6F TMI approach. In the dose junction region, the 93% of PTV was covered by the prescribed dose without obvious hotspots. For the 27 patients, the overall setup corrections were lower than 3 mm except those in the SI direction for abdomen-pelvis region, demonstrating excellent immobilization.

**Conclusion:**

The present study confirmed the technical feasibility of HT-based TMI/TMLI delivering 8-10Gy in 2 fractions over 1 day. For patients undergoing hematopoietic cell transplantation the proposed 8Gy/2F TMI (or 10Gy/2F TMLI) strategy may be a novel approach to improve delivery efficiency, increase effective radiation dose to target while maintaining low risk of severe organ toxicities.

## Background

Total body irradiation (TBI) has been an important part of conditioning regimens for patients undergoing hematopoietic cell transplantation [1]. The primary purpose of TBI is to eradicate malignant cells and provide immunosuppression to prevent rejection of the transplanted donor hematopoietic cells. Compared to the conditioning regimens based on chemotherapy alone, TBI has several distinct advantages because it is not influenced by interpatient variability in drug absorption, metabolism, biodistribution, or clearance kinetics; and can treat the sanctuary sites not easily reached by chemotherapy drugs. TBI also contributes to the elimination of chemotherapy-resistant tumor cells [2, 3].

Randomized trials showed that increased TBI doses significantly reduced the probability of post-transplant relapse rates for patients [4]. However, the dose escalation of TBI is limited by the normal tissue toxicity and treatment-related mortality rates [5, 6]. With traditional TBI delivery techniques, only lung blocks are used to reduce lung dose to some extent and no attempt is made to spare other organs at risk (OARs) such as the eyes, heart, liver, and kidney. As a result, acute and late complications of treatment may arise. Specifically, acute effects include nausea, vomiting, diarrhea, oral mucositis, parotitis and interstitial pneumonitis; long-term effects include cataracts, growth restriction, increased likelihood of heart disease and radiation-induced second malignancies. Given the fact that the incidence of radiation-induced complications is dose related [4–6], a more targeted irradiation technique for TBI delivery is needed to reduce normal tissue toxicity and allow for dose escalation, and thus further decrease mortality and relapsed rates.

Helical Tomotherapy (HT)-based total marrow (and lymphatic) irradiation (TMI-TMLI) may be one solution to optimize treatment and permit dose escalation [7–10]. Helical Tomotherapy system is a radiation therapy delivery device that equips a linear accelerator with a FAN beam mega-voltage computed tomography (MVCT) and a helical IMRT delivery, permitting the dose delivered to the target with maximum size of approximately 160 cm in length. HT allows greater sculpting of radiation doses to large complex target shapes while simultaneously reducing dose to normal organs, making it appropriate to be adopted for the delivery of TMI-TMLI.

The aim of the present study was to investigate the technical feasibility of HT-based TMI-TMLI, with the total prescription dose of 8 to 10Gy delivered by 2 fractions within one day with a minimal interfraction interval of 6 h. This report detailed the retrospective review of initial experience for patients undergoing HT-based TMI-TMLI and discussed the potential advantages and challenges of this approach. The evaluation of the TMI was also done by comparing the median organ doses with the conventional TBI and TMI reported by Wong et al. [10], in which 13 patients with multiple myeloma were treated.

## Methods

### Patient selection and simulation

Twenty-seven patients treated with TMI/TMLI using HT at our institution between October 2016 and September 2017 were selected for retrospective analysis. Majority of the patients included in the study were acute lymphoid leukemia (17), the rest were acute myeloid leukemia (6), multiple myeloma (2) and lymphoma (2). Of the 27 patients, 26 were adults and 1 was child. The mean and median age was 24.6 and 22 years (range 8–54), respectively. Nine patients received TMI while the others received TMLI. For the chemotherapy component of conditioning regimen, TMI/TMLI treatment (on day − 9) was followed by idarubicin 15 mg/m^2^/day for three consecutive days (days − 8 to − 6), then cyclophosphamide 30 mg/kg/day for 2 days (days − 4 to − 3) before transplant on day 0.

All patients underwent CT simulation using our departmental scanner (Sensation Cardiac 64x, Siemens, Munich, Bavaria, Germany). Because of the longitudinal scanning and treatment limit of the CT and HT treatment couch, two planning CTs, defined as upper body (CT_upper_) and lower limbs (CT_lower_), were acquired to cover the total patient cranial-caudal extension. The two CT scans with 5 mm slice thickness were collected in shallow free breathing mode to develop two corresponding treatment plans (Plan-upper and Plan-lower).

**Fig. 1:**
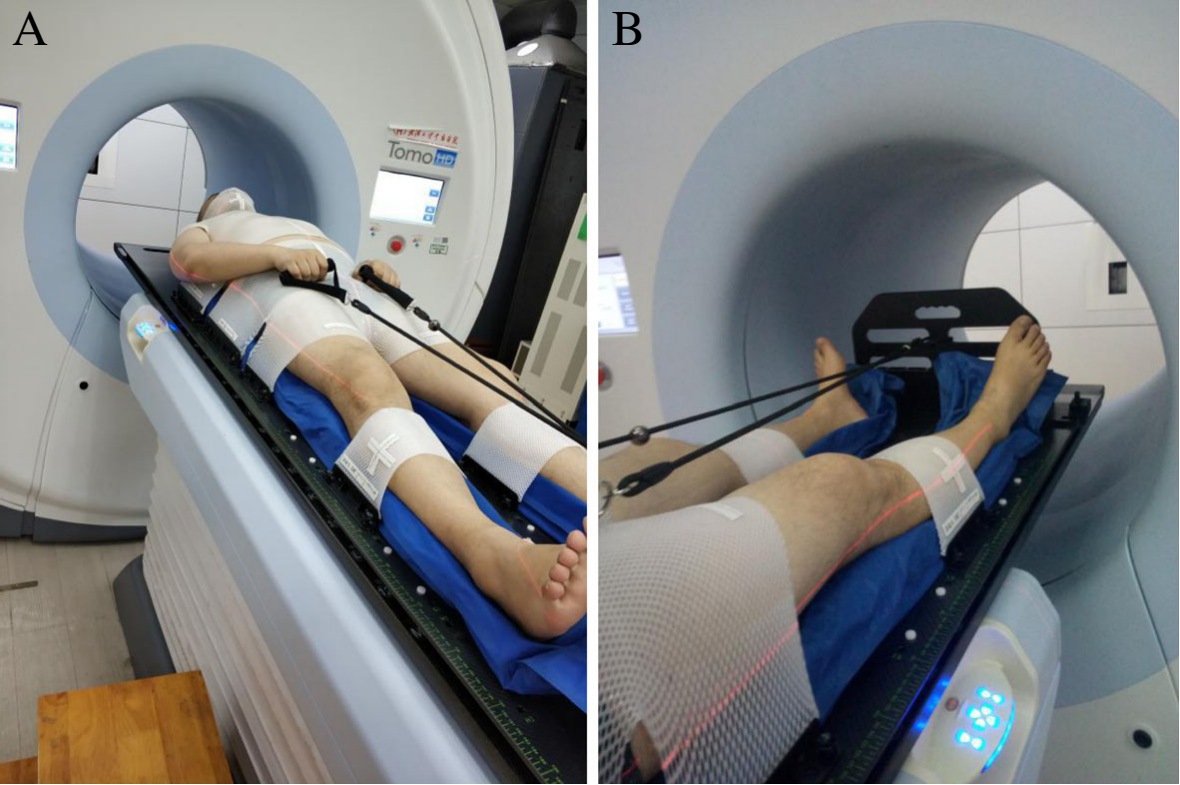
Example of the use of the 3 thermoplastic masks for patient immobilization. **a** head first supine orientation (HFS) and **b** feet-first in the supine position (FFS)

As is shown in Fig. 1, patients were positioned using a home-made dedicated immobilization system, which consisted of one all body frame, one integrated vacuum-formed cradle, one upper limb fixator and three personalized thermoplastic masks. Briefly, the first mask covered the head, neck, shoulder and chest, with arms closing to the thorax; the second immobilized the abdomen and pelvis while hands of the patients were positioned on the groins with fingers grasping the rope to ensure good reproducibility; the third immobilized the tibia to minimize lower limbs motion. Herein, three sets of markers were applied, among of which two sets were used for treatment positioning and alignment. The first set of fiducial markers was placed at the mandible level and the second was placed on the masks around the lower legs. And the third was placed 10 to 15 cm above the patient’s knees, in the lateral direction, as reference points to locate the overlapping region for dose junction (Fig. 5a). The rope length was customized to each individual patient. One end of the rope was fastened at the fixation device and the other end grasped by the patient. The shape and location of the hands were drawn on the thermoplastic mask to ensure reproducibility. To obtain CT_upper_, the patients were positioned in the head first supine orientation (HFS) (seen in Fig. 1a, with scan volume running from the vertex to the region closing to the knees. To acquire CT_lower_, the frame just made a U-turn with the support from two therapists so that the patients did not need to be let down from the couch and they were scanned feet-first in the supine position (FFS) (seen in Fig. 1b). The scans extended from the feet to the knees plus a margin of 15 cm to 20 cm in the cranial direction using the same immobilization device. Such a margin could be used to correctly account for dose junction.

### Target definition

**Fig. 2:**
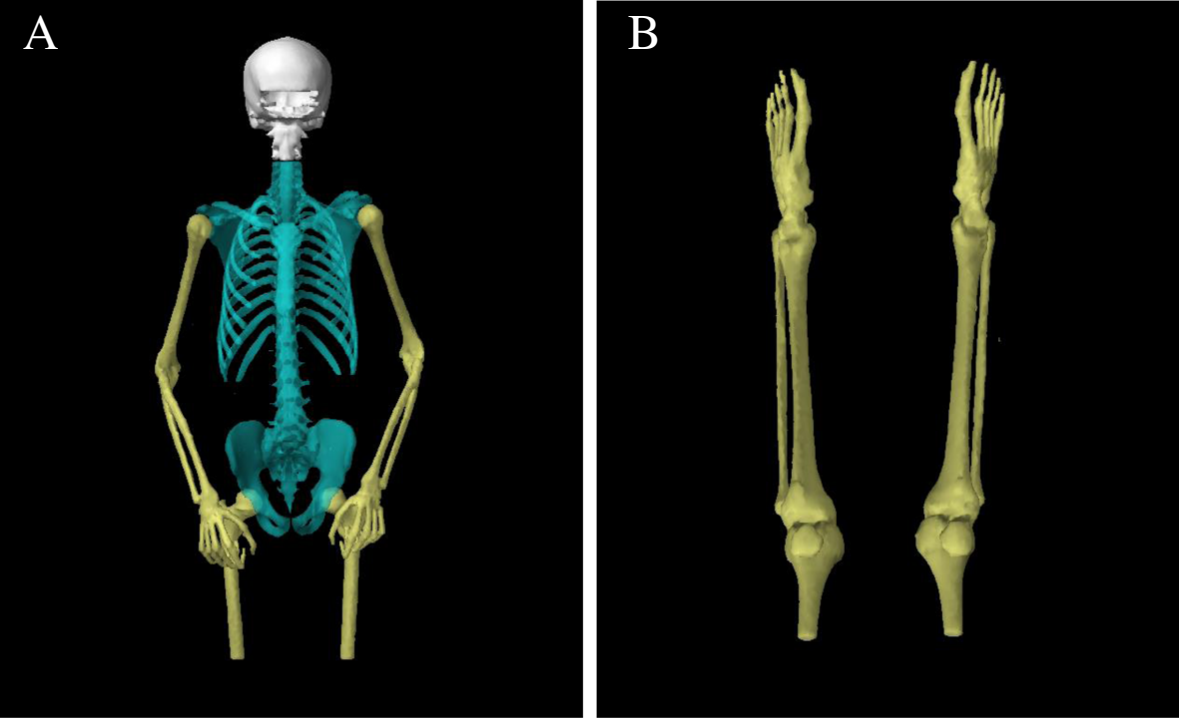
Typical CTV of total marrow irradiation including 1) head (white), 2) trunk (blue), 3) arms and legs (yellow). **a** CTV for Plan-upper and **b** CTV for Plan-lower

TMI is applicable as part of a conditioning regimen for multiple myeloma. For TMI, the clinical target volume (CTV) was defined as all skeletal bones exclusion of the mandible. Considering the possible involuntary motion and setup error, the CTV was divided into three subvolumes:head, trunk, arms and legs (Fig. 2). These three subvolumes were enlarged of 3, 5 and 10 mm in three dimensions respectively, to generate the planning treatment volume (called “PTV_bone_”). The OARs in the study included brain, brainstem, lens, eyes, optic nerves, parotids, oral cavity, lungs, heart, liver, kidneys, stomach, small bowel, bladder and rectum.

TMLI is applicable as part of a conditioning regimen for patients with acute lymphoid and myeloid leukemia. Herein, the target regions of skeletal bones (PTV_bone_) were contoured according with the aforementioned method as well as lymphatic sanctuary sites potentially including the major lymph node chains, liver, spleen, testes, and brain, plus additional margin of 5 mm in the three directions were included to generate PTV_lymph_ for the TMLI patients. Typically, mesenteric lymph nodes were not included in the PTV_lymph._

### Dose fractionation and plan optimization

For TMI patients, dose prescription to the PTV_bone_ was 8Gy in 2 fractions. For TMLI patients, 8Gy was prescribed to the PTV_bone_ and 10Gy was prescribed to PTV_lymph_. In both scenarios, the treatments were delivered in one day with a 6-h interval between fractions. The dose fractionation was chosen based on TDF model introduced by Supe SS et al. [11, 12] that 8Gy in 2 fractions BID was equivalent to 12Gy in 2Gy × 3 days BID.

$$ TDF=N{d}^{1.538}{\left(T/N\right)}^{-0.169}\times {10}^{-3} $$

where *d* is dose per fraction, *N* is the number of fractions and *T* is the total treatment time.

For 8Gy in 2 fractions BID, *N* = 2 fractions, *d* = 4Gy, *T* = 1 day; for 12Gy in 2Gy × 3 days BID, *N* = 6 fractions, *d* = 2Gy, *T* = 3 day. Results shown that 8Gy in 2 fractions BID was equivalent to 12Gy in 2Gy × 3 days BID. On the other hand, we also calculated the BED, with BED = *nd* (1 + *d*/(α/β)), where *d* is dose per fraction, *n* is number of fractions, and α/β is “intrinsic radiosensitivity,”. Herein, the α/β values used were 1.49 and 3.12, as suggested in samples from patients with acute myelogenous leukemia collected at the time of diagnosis [12]. The BED was approximately equal in the two dose fractionation schemes.

In the plan optimization process, the parameters were set as follows: 5 cm for the field width, pitch of 0.287 while modulation factor (MF) varied from Plan-upper to Plan-lower (shown in Table 1). Furthermore, due to the overlap region between CT_upper_ and CT_lower_, the dose homogeneous at the junction region from the contributions of the two plans should be considered. To minimize the dose inhomogeneity, the PTVs from the upper plan were optimized prescribing, 5Gy, 4Gy and 3Gy, respectively, in the three consecutive slices adjacent to the dose junction region. Similarly, the isodoses of 3Gy, 4Gy and 5Gy from the lower plan were prescribed on the CT_lower_, to complement the dose distributions of Plan-upper (Fig. 5a). In this way a plan sum in the field junction region without creating cold spots and hotspots was produced. The MIM© software (Cleveland, Ohio) was used to generate the sum plan and evaluate dose Inhomogeneity.

**Table 1:** The length of the PTV, MF and the beam-on time for upper and lower body treatment plans over the 27 patients

	The length of the PTV(cm)	MF	The beam-on time(min)
Plan-upper			
Mean	110.8	2.9	46.1
Range	90.9–112.9	2.7–3	36.4–55.8
Plan-lower			
Mean	71.0	1.7	16.3
Range	57.2–81.3	1.4–2	13.0–19.6

Abbreviations: *PTV* Planning target volume, *MF* Modulation factor

All plans were generated adopting an identical set of PTV/OAR dose–volume constraints. The criterion for acceptance of the plan was that at least 90% of the PTV received the prescription dose [13, 14], with the primary objective being to reduce the normal organ dose to a minimum.

Data from the dose volume histogram (DVH) acquired for all contoured organs and the target volumes was analyzed. For the OARs, a set of dosimetric parameters was obtained, including the mean dose (D_mean_), the maximal dose (D_max_), the V_2Gy_ (the percent of volume that received *2*Gy), V_4Gy_, V_6Gy_, and V_8Gy_. For the PTV, D_mean_, D_max_, V_7Gy_, V_8Gy_, V_9Gy_, V_10Gy_ and V_11Gy_ were quantified.

### IGRT and dose delivery

Considering the long TMI/TMLI beam-on time and organ/patient motion, four MVCT scans for each patient were obtained (three for the Plan-Upper delivery and one for Plan-Lower) in order to check the patient’s whole body alignment. An automatic registration process of the kilovoltage CT/MVCT fusion was performed utilized three rigid translations in the left–right (LR), superior–inferior (SI), and anterior–posterior (AP) directions, as well as roll (rotation around the SI axis). After the automatic image registration, the attending physician verified the image fusion and alignment to ensure proper alignment of the PTV region.

For the treatment of upper body, the first scan ranged from orbits to the first cervical vertebra. After image registration, treatment was started after alignment shift. When treatment approached the end of the first MVCT, the treatment was manually interrupted and a second MVCT scan (including part of the lung volume) was performed. After the image registration, the shifts in LR/AP/roll directions were applied to patient’s alignment while the shifts in SI direction were set to zero in order to avoid hotspots around the field junctions, then resume treating. As before, when treatment approached the end of the second MVCT, the treatment was manually stopped again and a third MVCT (from the kidneys to the pelvic region) was scanned. The shifts in SI direction were set to zero and the shifts in other directions were applied to line up the specific MVCT. Therefore, the treatment of the upper body was manually interrupted twice.

For the treatment of the lower part of the body, a fourth MVCT scan (the knee-joint region) was performed to check patient alignment. Despite well immobilized, patients were still closely monitored for any noticeable movement by the therapist using the in-room video cameras, especially for the last treatment because of the possible patient discomfort or nausea.

## Results

### Treatment parameters

Table 1 listed the length of the PTV, the modulation factor (MF) and the beam-on time (BOT). Specifically, the MF is the one that we set up before the calculation of the plan, instead of the “effective” one after the last calculation. Compared to Plan-lower, the MF of Plan-upper was relatively higher, ranged from 2.7 to 3. Increasing the modulation factor increased the target dose conformity and organ sparing at expense of longer treatment time. Therefore, the high MF for Plan-upper and the low MF for Plan-lower were used to keep the beam-on time as short as possible while maintaining the dose distribution acceptable. Correspondingly, the average beam-on time for Plan-upper was approximately 46 min (range: 36–56 min); it was 16 min (range: 13–20 min) for Plan-lower. The total time of TMI/TMLI treatment was about 2.5 h because of the necessity for acquiring and processing the MVCT scans.

### Target coverage and OAR sparing

**Fig. 3:**
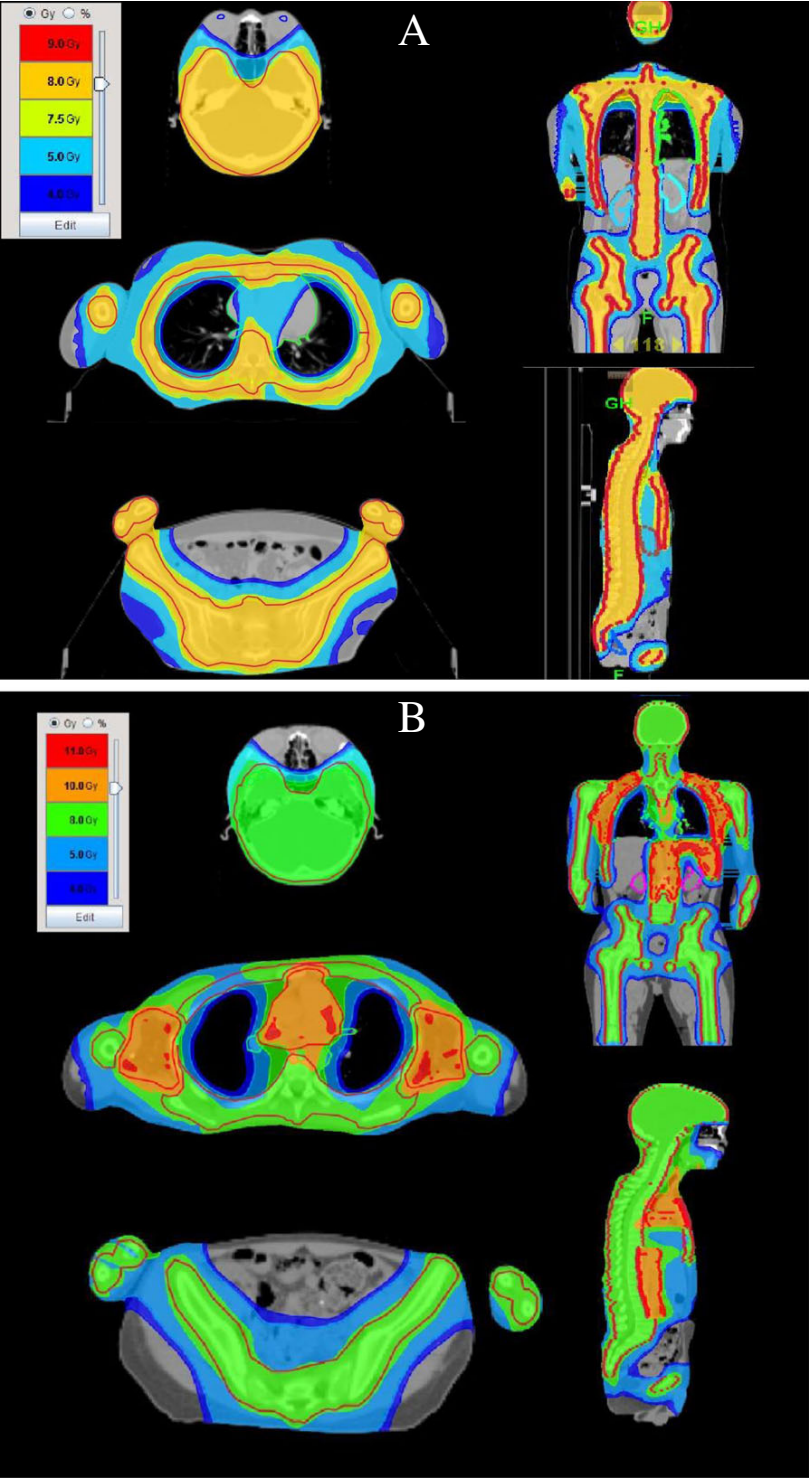
Color wash demonstrating dose distributions of a typical TMI/TMLI plan. **a** TMI; **b** TMLI

**Fig. 4:**
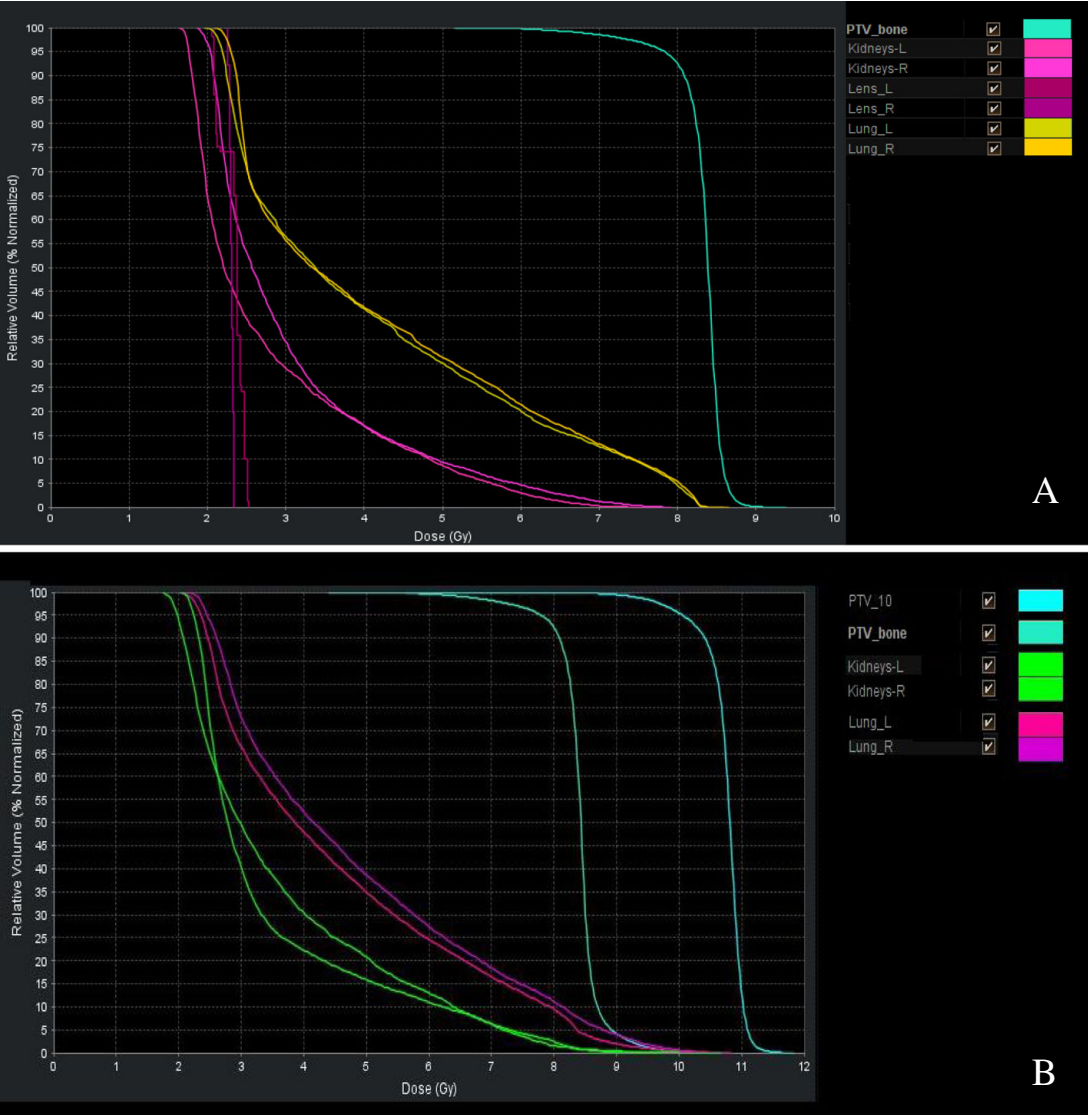
DVH curves of major organ and PTV of the typical TMI/TMLI plan. **a** TMI; **b** TMLI

Figure 3 showed the dose distribution of TMI and TMLI, demonstrating the successful sculpting of the prescription dose to the target with avoidance of lungs, lens, kidneys and other normal tissues. Figure 4 displayed the DVH of the same patients. The separation between the PTV and OAR dose–volume histograms indicated the successful sparing of the major normal organs.

Table 2 summarized the DVH parameters of PTV. In TMI patient cohort, 92% of the PTV_bone_ received the prescription dose of 8Gy. The average D_mean_ and D_max_ were 8.36Gy (104.5% of the prescription dose) and 9.76Gy (122%), respectively. In TMLI patient cohort, 91% of the PTV_lymph_ received the planned dose of 10Gy while 96% of the PTV_bone_ received 8Gy. The average D_mean_ and D_max_ of PTV_lymph_ were 10.36Gy and 11.39Gy, respectively. The plan acceptance criterion used in the present study for PTV was at least 90% of the PTV received the prescription dose.

**Table 2:** DVH analysis for the PTV

		V_7Gy_	V_8Gy_	V_9Gy_	V_10Gy_	V_11Gy_	D_mean_ (Gy)	D_max_ (Gy)
TMI	PTV_bone_	0.99 ± 0.00	0.92 ± 0.01	0.00 ± 0.00	–	–	8.36 ± 0.14	9.76 ± 0.22
TMLI	PTV_bone_	1 ± 0	0.96 ± 0.01	0.91 ± 0.03	0.18 ± 0.13	0.1 ± 0.11	8.61 ± 0.30	10.82 ± 0.76
	PTV_lymph_	1.00 ± 0.00	0.99 ± 0.01	0.92 ± 0.26	0.91 ± 0.05	0.01 ± 0.01	10.36 ± 0.11	11.39 ± 0.39

Abbreviations: *PTV* Planning target volume, *DVH* Dose volume histogram, *TMI/TMLI* Total marrow/lymphoid irradiation, *V*_*xGy*_ (the percent of volume that received *x* Gy), *D*_*mean*_ The mean dose, *D*_*max*_ The max dose

Tables 3 and 4 listed the quantitative doses for selected organs for TMI and TMLI, respectively. For all TMI patients, the mean doses of the OARs were approximately 30–65% of the prescribed PTV dose, rarely exceeding 5Gy, except for the doses to the optic nerves, for which the average dose was about 5.2Gy. The lenses, with an average max dose of approximately 2.6Gy, were the organs that received the least dose. Compared to the lower OARs doses of TMI, the mean dose of kidneys, heart and liver in TMLI patients increased as expected, approximately 63–89% of the prescribed 10Gy to PTV_lymph_, due to the radiation to the major lymph node chains, liver, spleen and brain.

**Table 3:** Organs at risk doses in Gy for 9 TMI patients

Organs	V_2Gy_	V_4Gy_	V_6Gy_	V_8Gy_	D_mean_ (Gy)	D_max_ (Gy)
Lung Left	0.96 ± 0.09	0.48 ± 0.06	0.23 ± 0.06	0.06 ± 0.06	4.77 ± 0.33	9.16 ± 0.84
Lung Right	0.97 ± 0.04	0.47 ± 0.06	0.23 ± 0.06	0.06 ± 0.06	4.62 ± 0.31	8.67 ± 0.34
Eye Right	0.84 ± 0.17	0.23 ± 0.15	0.04 ± 0.05	0 ± 0	3.18 ± 0.71	5.34 ± 0.93
Eye Left	0.81 ± 0.16	0.21 ± 0.13	0.02 ± 0.02	0 ± 0	3.27 ± 0.65	5.66 ± 1.22
Lens Right	0.78 ± 0.42	0.07 ± 0.12	0 ± 0	0 ± 0	2.43 ± 0.58	2.86 ± 0.56
Lens Left	0.7 ± 0.38	0.02 ± 0.05	0 ± 0	0 ± 0	2.42 ± 0.61	2.79 ± 0.64
Optic nerve Right	1 ± 0	0.49 ± 0.3	0.22 ± 0.36	0 ± 0	5.15 ± 1.77	6.4 ± 1.84
Optic nerve Left	0.92 ± 0.17	0.63 ± 0.4	0.31 ± 0.31	0 ± 0	5.26 ± 1.51	6.74 ± 1.51
Kidney Left	0.98 ± 0.03	0.28 ± 0.1	0.1 ± 0.05	0.01 ± 0.01	3.41 ± 0.77	7.96 ± 1.17
Kidney Right	0.98 ± 0.03	0.25 ± 0.14	0.08 ± 0.08	0.01 ± 0.02	3 ± 0.14	7.42 ± 0.5
Heart	1 ± 0	0.57 ± 0.13	0.29 ± 0.1	0.06 ± 0.07	4.65 ± 0.51	8.69 ± 0.57
Liver	1 ± 0	0.63 ± 0.16	0.33 ± 0.27	0.17 ± 0.34	4.54 ± 0.37	8.93 ± 0.67
Small Bowel	0.94 ± 0.07	0.51 ± 0.21	0.13 ± 0.08	0.01 ± 0.01	4.26 ± 0.5	8.53 ± 0.81
Stomach	0.89 ± 0.18	0.51 ± 0.42	0.35 ± 0.38	0.17 ± 0.19	4.12 ± 0.94	7.73 ± 1.57

Abbreviations: *TMI* Total marrow irradiation, *V*_*xGy*_ (the percent of volume that received *x* Gy), *D*_*mean*_ The mean dose, *D*_*max*_ The max dose

**Table 4:** Organs at risk doses in Gy for 18 TMLI patients

Organs	V_2Gy_	V_4Gy_	V_6Gy_	V_8Gy_	D_mean_ (Gy)	D_max_ (Gy)
Lung Left	0.99 ± 0.03	0.52 ± 0.09	0.27 ± 0.09	0.04 ± 0.05	4.34 ± 0.49	9.54 ± 0.91
Lung Right	0.99 ± 0.02	0.51 ± 0.08	0.26 ± 0.08	0.03 ± 0.04	4.38 ± 0.46	9.44 ± 0.91
Eye Right	0.83 ± 0.26	0.32 ± 0.25	0.06 ± 0.11	0.01 ± 0.02	3.45 ± 0.99	6.72 ± 1.68
Eye Left	0.83 ± 0.24	0.3 ± 0.2	0.05 ± 0.08	0 ± 0	3.27 ± 0.91	6.33 ± 1.46
Lens Right	0.73 ± 0.39	0.03 ± 0.13	0 ± 0	0 ± 0	2.31 ± 0.8	2.76 ± 1.15
Lens Left	0.67 ± 0.45	0 ± 0	0 ± 0	0 ± 0	2.19 ± 0.71	2.52 ± 0.86
Optic nerve Right	0.99 ± 0.02	0.74 ± 0.36	0.56 ± 0.4	0.07 ± 0.13	5.38 ± 1.43	6.96 ± 1.03
Optic nerve Left	0.99 ± 0.03	0.78 ± 0.32	0.53 ± 0.4	0.04 ± 0.09	5.25 ± 1.63	6.83 ± 1.54
Kidney Left	0.93 ± 0.09	0.87 ± 0.18	0.8 ± 0.27	0.67 ± 0.44	8.86 ± 2.21	10.18 ± 1.1
Kidney Right	0.9 ± 0.11	0.78 ± 0.23	0.64 ± 0.36	0.55 ± 0.45	6.16 ± 2.27	7.93 ± 0.97
Heart	0.96 ± 0.09	0.83 ± 0.33	0.41 ± 0.3	0 ± 0	6.34 ± 0	8.05 ± 0
Liver	0.98 ± 0.05	0.81 ± 0.36	0.38 ± 0.32	0.02 ± 0.03	6.88 ± 0	8.23 ± 0
Small Bowel	1 ± 0	0.6 ± 0.12	0.28 ± 0.11	0.03 ± 0.05	5.01 ± 0.47	8.96 ± 0.85
Stomach	0.99 ± 0.01	0.57 ± 0.14	0.25 ± 0.19	0.08 ± 0.21	5.64 ± 2.19	9.51 ± 1.27

Abbreviations: *TMLI* Total marrow and lymphoid irradiation, *V*_*xGy*_ (the percent of volume that received *x* Gy), *D*_*mean*_ The mean dose, *D*_*max*_ The max dose

The median OAR doses were then compared with that of conventional TBI previously reported by Wong et al. and that of TMI [9, 10] in Table 5. On average, the median dose of the kidneys decreased by 65 and 24% compared with that of TBI and TMI, respectively. Additional reduction in the median lung doses could be achieved only by compromising the target dose coverage in the chest region. Please note that there were difference in CTV delineation and PTV margin in different studies. The direct absolute dose comparison may not be applicable. However, it was at least demonstrated that the current study achieved similar or better dose distributions.

**Table 5:** The median doses of organs at risk in Gy (%, normalized to their respective prescription dose) for TBI (12Gy), TMI(12Gy) and TMI(8Gy)

Organs	TBI 12Gy	TMI 12Gy	TMI 8Gy
Lens	11.3Gy (94.17%)	1.7Gy (14.17%)	2.58Gy (32.25%)
Lungs	8.8Gy (73.33%)	5.9Gy (49.17%)	3.84Gy (48%)
Kidneys	12.2Gy (101.67%)	7.2Gy (60%)	2.88Gy (36%)
Heart	12.1Gy (100.83%)	6Gy (50%)	4.6Gy (57.5%)
Liver	12.6Gy (105%)	7.5Gy (62.5%)	4.73Gy (59.13%)
Eyes	11.3Gy (94.17%)	6Gy (50%)	2.88Gy (36%)
Bowel	12.3Gy (102.5%)	4.8Gy (40%)	3.95Gy (49.38%)
Stomach	12.2Gy (101.67%)	4.6Gy (38.33%)	4.1Gy (51.25%)

Abbreviations: *TBI* Total body irradiation, *TMI/TMLI* Total marrow/lymphoid irradiation

### Dose evaluation in the overlapping area

**Fig. 5:**
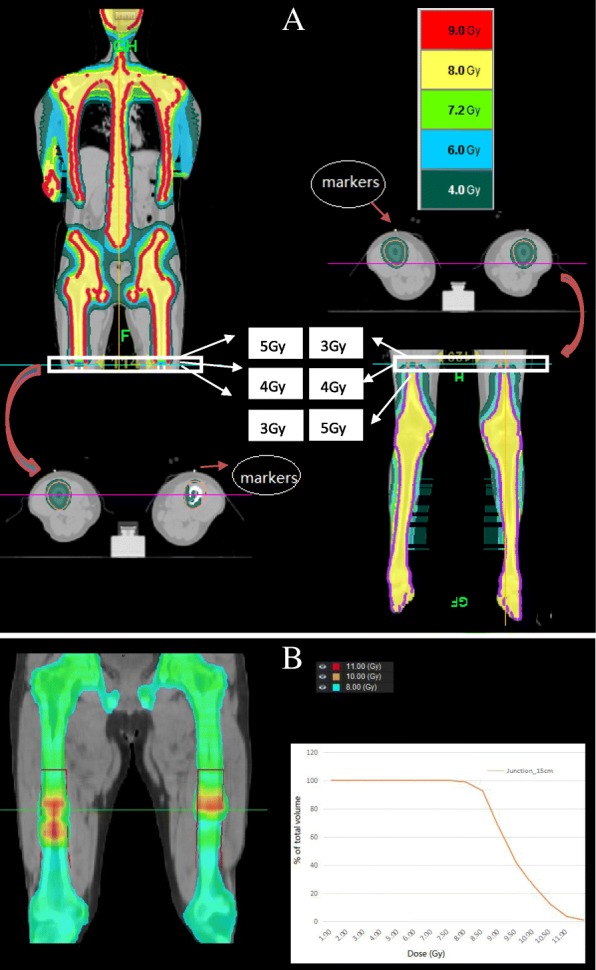
Details of dose distributions in junction region of for the patient above, including the dose color wash and the DVH. **a** dose distributions of Plan-upper(left) and Plan-lower(right); **b** plan summation and the corresponding DVH in the junction region

To appreciate the magnitude of the field junction problem, a typical DVH arising from the plan sum and the corresponding dose distribution is shown in Fig. 5b by virtue of the MIM software. In the overlap region of 15 cm, the doses ranged from 6.75Gy to 11.19Gy, with the mean dose being 8.95Gy. As seen in Fig. 5, the 93% of the target volume in the abutting region managed to be covered by the prescribed dose. No obvious hot or cold spots in the field junctions were noticed.

### Setup errors during treatment

Table 6 showed statistics of patients setup errors obtained by the MVCT scan registered with the planning CT scan. Precise setup of the patients was necessary for any IMRT case and only a 5-mm difference between the two scans in the three translation directions and 1° of difference in roll were allowed to perform the treatment. For the 27 patients, the overall setup corrections were 1.06 ± 0.79 mm in the LR direction, 1.34 ± 0.66 mm in the SI direction, and 2.45 ± 1.48 mm in the AP direction and 0.63 ± 0.65° in roll for head and neck region. The setup corrections in other region were also small, demonstrating the effectiveness of the current immobilization technique.

**Table 6:** Setup errors of patients underwent TMI/TMLI

	LR (mm)	SI (mm)	AP (mm)	Roll (°)
Head-neck	1.06 ± 0.79	1.34 ± 0.66	2.45 ± 1.48	0.63 ± 0.65
Thorax	1.58 ± 1.13	2.38 ± 1.99	2.05 ± 1.68	0.31 ± 0.32
Abdomen-pelvis	1.67 ± 1.24	3.88 ± 2.20	1.96 ± 1.32	0.48 ± 0.53
Legs	0.95 ± 0.73	1.99 ± 1.35	3.66 ± 2.13	0.24 ± 0.31

Abbreviations: *TMI/TMLI* Total marrow/lymphoid irradiation, *LR* Left–right, *SI* Superior–inferior, *AP* Anterior–posterior, *Roll* Rotation around the SI axis

## Discussion

Recently, TMI-TMLI has been explored as bone marrow transplantation conditioning regimens. Compared with the conventional TBI, the dosimetric studies with TMI-TMLI demonstrated the increased dose conformity to total marrow or lymphoid tissues and the decreased doses to normal organs which predicted for reduced toxicities. However, the clinical experiences on TMI are still limited with a low number of patients treated so far. In our institute since 2016, TMI-TMLI was delivered by Helical Tomotherapy using 8-10Gy in 2 fractions over one day. Different from the typical 12Gy TBI/TMI schedule (i.e. total dose of 12Gy, 2Gy per fraction, twice per day) [15–17], in this feasibility study we increased the fractionated dose to the target. The rationale we followed to explore this novel fractionated TMI approach was based on the several reasons. (1) It is known that the biological effect of the physical dose depends on the radiobiological characteristics of the relevant tissue, fractionation scheme, dose rate and treatment time. Thus, the absorbed dose needs to be translated into a biological equivalent dose to predict the biological effect of radiotherapy treatments. With decades of clinical experience, various models including the time dose fractionation (TDF) formula have been obtained and widely used to estimate the effects of fractionation. According to TDF formula, the biological equivalent dose of 8Gy/2F approximates that of 12Gy/6F [11, 12]. (2) The proposed TMI strategy will facilitate the treatment efficiency. Although TMI is effective for patients with acute leukemia, the traditional TMI treatment is very time consuming, generally delivering 12Gy over 3 days (about 2 h per treatment session, twice daily, 6 sessions in total). And increasing the dose of TMI to 16–20Gy will further increase the dose delivery time [10, 15–18]. The proposed 8Gy/2F TMI approach could be accomplished on 1 day and streamline the treatment process significantly. (3) More importantly, clinical efforts suggest that modest increases in TBI dose could result in decreased relapsed rates, but such treatment modality is limited due to the high risk of increased toxicity mainly to the lungs, liver, and kidneys. Since the HT-based TMI delivers highly conforming dose distributions selectively to bone marrow, TMI treatment has the potential to decrease toxicities and permit dose escalation to tumors. Whether the dose escalation should be implemented by increasing dose per fraction or by increasing number of fractions remains to be answered, potentially by clinical trials. Several groups have attempted to escalate TMI doses in an effort to improve outcomes. For example, previous publications have demonstrated that it is possible to increase the dose of TMI with a multistep process up to 16–20Gy with the potential of a reduced risk of radiation-related toxicity [10, 15–18]. Additionally, some trials increased radiation dose to the marrow by adding 2Gy TMI after the delivery of a conventional 12Gy TBI schedule [19]. Other researchers delivered the prescribed dose (3Gy per fraction in 5 or 6 fractions) to the bone marrow [12] to reduce the relapsed rates. In this report, we present the preliminary data obtained exploring the technical feasibility of TMI/TMLI strategy with increased fractionated dose (4Gy per fraction). No acute adverse effects occurred during the treatment and only two patients suffered nausea or vomiting right after radiation course. As expected with TMI, the median dose reduction of major organs varied 30–65% of the prescribed dose, substantially lower than the traditional TBI and predicting for reduced acute toxicities. Furthermore, average doses to OARs with 8Gy/2F TMI approach was not different from the conventional 12Gy/6F TMI approach, providing a compelling reason to evaluate the clinical utility of this novel fractionation scheme. In fact, the proposed TMI/TMLI strategy with 4Gy per fraction allows for further dose escalation to target, especially to the lymphoid tissues. Nevertheless, it should be noted that adjustment of both treatment parameters and dose objectives are required to ensure more rigorous organ sparing if a dose escalation protocol is to be adopted. Overall, such TMI delivery approach consisting of a novel dose fractionation strategy appears interesting in preliminary clinical results and deserves further investigation.

In this context, the dose on the junction area from the contributions of the two plans should be properly handled to minimize the inhomogeneity and avoid hot or cold doses [20–22], Herein, both the mean dose (8.95Gy) and max dose (11.19Gy) of the junction region were greater than the prescription dose, similar to those reported by Zeverino [19]. There may be room of improvement for the dose homogeneity. However, due to the fact that the CT scans were acquired with 5-mm slice thickness and it is not possible to optimize one plan based on the other in two different CTs in HT, the homogeneity of dose distribution within the junction seemed not as good as what would be expected. Further research can be conducted to evaluate the robustness of the junction simulating patient shift and explore optimal strategy to obtain homogeneous dose on the junction region.

Some uncertainties related to the TMI/TMLI treatment remain to be answered. For instance, instantaneous dose rates for HT TMI can be as high as 8Gy/min, far in excess of the typical TBI dose rates, which generally ranged from 0.05 to 0.5Gy/min. For single-session TBI, the low dose rate would reduce the complications [23], but this effect does not exist or is greatly diminished for fractionated treatment [9, 24]. The impact of the higher radiation dose rate in HT on complications and engraftment remains to be determined. Another uncertainty is related to organ motion. The CT scans, as described, were collected in shallow free breathing mode and no PRVs (planning organs at risk volume) created were for the OARs (especially lung, kidney, other organs that move with breathing). Considering the long TMI/TMLI beam-on time and organ/patient motion, the irradiation was segmented in four parts (three for the Plan-Upper delivery and one for Plan-Lower) [14, 25] while four MVCT scans for each patient were also obtained in order to check the patient’s whole body alignment.

Previous studies and the present work have demonstrated that the normal tissue doses responsible for the sequelae are significantly reduced using HT-based TMI/TMLI. However, the developmental abnormalities associated with skeleton in pediatric cases may be one question worth exploring. Given there was only one pediatric case in this study, there was not enough data to suggest any modification to target definition.

## Conclusions

The present work exhibited the technical feasibility of HT-based TMI/TMLI delivering 8-10Gy in 2 fractions over 1 day, suggesting the potential of the novel dose fractionation strategy in improving the treatment efficiency, potentially outcome and therapeutic efficacy for patients undergoing hematopoietic cell transplantation with low risk of severe toxicity.

## Abbreviations

AP: Anterior–posterior
CT: Computed tomography
CTV: Clinical target volume
FFS: Feet-first in the supine position
HFS: Head first supine orientation
HT: Helical tomotherapy
LR: Left–right
OAR: Organs at risk
PTV: Planning target volume
SI: Superior–inferior
TBI: Total body irradiation
TDF: Time dose fractionation
TMI: Total marrow irradiation
TMI-TMLI: Total marrow (and lymphatic) irradiation
TMLI: Total marrow and lymphatic irradiation
V_*x*Gy_: The percent of the tumor volume that received *x* Gy
